# Cooperative Navigation for Cross-Platform Dual-SINS Based on Relative Range and Angle Measurements

**DOI:** 10.3390/s26144450

**Published:** 2026-07-13

**Authors:** Jiang Lai, Shiqiao Qin, Xiangyuan Li, Jiaxing Zheng, Wenfeng Tan, Yingwei Zhao

**Affiliations:** College of Advanced Interdisciplinary Studies, National University of Defense Technology, Changsha 410073, China; resiro@163.com (J.L.); sqqin8@nudt.edu.cn (S.Q.); busybeelxy@163.com (X.L.); zhengjiaxing@nudt.edu.cn (J.Z.); tanwenfeng08@nudt.edu.cn (W.T.)

**Keywords:** cooperative navigation, observability analysis, bias estimation, information fusion, path planning

## Abstract

**Highlights:**

**What are the main findings?**

- A cooperative navigation method for dual-SINS is proposed using only relative range and angle measurements, enabling full-bias estimation with simple planar motion.
- Experimental results show that after 3 h of cooperative navigation along a figure-8 trajectory, the positioning accuracy of the two SINSs improves by 77.4% and 68.4% compared to autonomous mode.

**What are the implications of the main findings?**

- Based on relative range and angle measurements, the proposed method eliminates the need for high-precision reference benchmarks, complex 3D excitation trajectories, or turntable modulation.
- It reduces system complexity and motion requirements, providing an effective and easy-to-implement solution for GNSS-denied cooperative navigation, especially for UGVs.

**Abstract:**

In order to address the issue of rapidly divergent positioning errors of a single-platform inertial navigation system (INS) in GNSS-denied environments, this paper proposes a cross-platform cooperative navigation method based on relative range and angle measurements. The observability of the cooperative navigation system under different motion strategies is investigated using Fisher information matrix (FIM) right null-space analysis combined with singular value decomposition (SVD). The results show that with relative range and angle measurement constraints, all inertial sensor biases can be effectively estimated by two strapdown inertial navigation systems (SINSs) moving along a simple trajectory, thereby improving the navigation accuracy. Experimental results demonstrate that compared to the autonomous navigation mode, the average positioning accuracy of the two SINSs improves by 77.4% and 68.4% respectively after 3 h of cooperative navigation along the prescribed trajectory. Using relative range and angle measurements, the proposed method requires only two SINSs and relatively simple planar motion, without the need for high-precision reference benchmarks, complex three-dimensional excitation trajectories, or turntable modulation. It reduces system complexity and motion requirements, providing an effective and easy-to-implement solution for ground vehicular positioning and orientation and other cross-platform cooperative navigation tasks in GNSS-denied environments.

## 1. Introduction

With the increasing complexity of navigation tasks and the dynamic changes in navigation scenarios, GNSS signals face the challenges of unreliability or even unavailability in many scenarios. Relying solely on a single-platform sensor makes it difficult to obtain higher-accuracy positioning and navigation results [1,2,3,4]. Introducing motion or displacement constraints is a widely adopted approach to suppress error accumulation in GNSS-denied environments. For example, in downhole wellbore surveying, a dead reckoning method utilizing known drill pipe length as a displacement constraint effectively improves trajectory tracking accuracy in magnetic interference environments [5]. However, such single-platform constraint methods can only optimize performance based on the carrier’s own motion information and can hardly break through the accuracy ceiling determined by the inherent error characteristics of the inertial sensor.

To address this inherent limitation, introducing inter-platform relative measurements and cooperative processing provides a viable path. Enhancing the overall navigation performance through information exchange and cooperative processing among multi-platform INSs by exploiting relative measurements and geometric constraints between platforms is an effective way to break through the performance bottleneck of a single platform and achieve high-accuracy navigation [6,7]. In the existing literature, such methods are commonly referred to as collaborative navigation or cooperative navigation [8].

The difference between collaborative navigation and cooperative navigation lies in whether there exists a platform that can independently calculate its own accurate position. Collaborative navigation typically assumes that at least one platform is always under GNSS coverage and can obtain high-accuracy positioning results, and then uses relative measurements to build constraints with other platforms, thereby improving the overall positioning accuracy of the swarm [9,10,11,12]. Alternatively, it may directly use a high-precision INS as a reference to improve the positioning accuracy of low-cost INSs through relative measurements [13]. Cooperative navigation, on the other hand, does not rely on any external positioning reference or high-precision sensors. It generally assumes that each platform cannot determine its own position with sufficient accuracy and only uses inter-platform relative measurements and geometric constraints to mutually correct the estimations, thereby improving the overall positioning accuracy. In other words, collaborative navigation relies on the propagation of an absolute position reference, while cooperative navigation completely depends on constraints from relative measurements.

In [6], the importance of an absolute position reference is theoretically demonstrated: as long as at least one platform in the swarm can receive GNSS signals and can establish communication and relative range measurements with other platforms, the absolute positions and yaw angles of all platforms become observable. The “father-son” framework in [9,10] effectively validates this conclusion, where the “father” vehicle operates in a GNSS-covered area and the “son” vehicle operates in a GNSS-denied environment. The “father” vehicle establishes relative positional relationships with the “son” vehicle through visual tracking and relative ranging, thereby assisting the “son” vehicle in determining its own position.

In a fully GNSS-denied environment where no platform can provide an absolute position reference, cooperative navigation relies solely on relative measurements, and the system observability suffers from an inherent deficiency—the absolute positions and yaw angles of all platforms are unobservable [14]. This leads to accumulated estimation errors over time and severely affects the reliability of long-duration missions. In [15], it is pointed out that under the condition of no absolute position reference, performing a series of maneuvers to achieve a certain formation geometry, combined with relative measurements constraints between platforms, can effectively improve the observability of cooperative navigation systems. In [16], the Lie derivative method is used to analyze the range-only cooperative navigation system, and observability criteria for trajectory design are derived: the relative position vector between the two INSs must not be constant in direction and distance, and a single platform must not move along a straight-line trajectory; otherwise, states such as relative position and gyro biases become unobservable. In [17], the unobservable directions of a dual-IMU system under special motion conditions (e.g., stationary, straight-line, and planar motions) are further analyzed. Such is the improvement in relative positioning accuracy achieved by [18,19,20]; we built upon these works, through trajectory design using methods such as Fisher information matrix, geometric dilution of precision (GDOP), and observability Gramian, respectively. However, all the above methods aim to optimize relative positioning accuracy and do not consider how to improve absolute positioning accuracy.

Due to the existence of gyro and accelerometer biases as well as random noises, navigation errors of an INS diverge rapidly over time [21,22,23]. Biases estimation is beneficial for the absolute positioning accuracy improvement [16], among which the estimation of the vertical gyro bias has the most significant impact on positioning errors. To make the vertical gyro bias observable, existing cooperative navigation methods often require complex hardware configurations or excitation motions.

In [24], multiple single-axis rotational INSs were employed, and the vertical gyro bias is made observable by exploiting the AUV’s yaw maneuvers and the lever-arm effect between the INSs. Although this strategy imposes relatively mild demands on vehicle dynamics, it still requires additional rotating mechanisms and specific yaw motions, limiting its versatility under constrained conditions. In [25], four MEMS-IMUs were orthogonally placed, and their biases are estimated through rotation modulation and redundant configuration without external excitation. While this design can achieve self-calibration under benign carrier motion, the increased number of sensors, precise orthogonal installation, and algorithmic complexity may restrict its use in compact or cost-sensitive platforms. In [26], an adaptive trace cost function generated trajectories that minimize bias covariance, and three-dimensional motions excite all six IMU axes for bias convergence. This approach requires no additional hardware, but relies entirely on complex three-dimensional excitation trajectories, which are impractical for platforms that primarily perform planar motions.

To reduce system complexity and the need for motion excitation while improving the absolute positioning accuracy of the involved SINSs, this paper proposes a cooperative navigation method for cross-platform SINSs aided by a relative ranging and angle measurement device (RAMD). Using the constraints of relative range and angle measurements, a cooperative navigation filter equation between two SINSs is established. Without relying on any absolute reference, the proposed method achieves online estimation of all biases through simple planar motion, effectively improving the navigation accuracy of the involved SINSs. The main contributions of this paper are as follows:

1. Based on the FIM right null-space analysis combined with SVD, the observability of a dual-SINS system under different motion patterns is analyzed with relative range and angle measurement constraints. The correlations among different state variables are revealed, and sufficient motion conditions for making key system states (particularly the z-axis gyro bias) observable are provided.
2. To achieve reliable cooperative navigation, a cooperative navigation model based on relative range and angle measurement is established. A simple planar motion pattern is proposed to achieve effective estimation of all biases of the two SINSs. Simulations are conducted to verify the effectiveness of the proposed method.
3. To further validate the effectiveness and robustness of the proposed algorithm, a corresponding cooperative navigation framework is designed, and an experimental platform is built. The experimental results confirm the effectiveness of the proposed method under realistic operating conditions.

The remainder of this paper is organized as follows. Section 2 presents the cooperative navigation model based on relative range and angle measurements. Section 3 analyzes the observability of the cooperative navigation system under different motion patterns using FIM. Section 4 and Section 5 describe the simulation and field experiment results, respectively. Finally, Section 6 concludes the paper and discusses future work.

## 2. Cooperative Navigation Model

### 2.1. Coordinate Frame Definition

The coordinate frames employed in this manuscript are listed in Table 1.

The transformation between coordinate frames can be represented by a direction cosine matrix (DCM). The DCM from the n frame to the $n^{\prime }$ frame $C_{n}^{n^{\prime }}$ can be derived through the cosine matrices multiplication [27]:

(1) $$C_{n}^{n^{\prime }}=\left[\begin{array}{ccc} \cos\phi_{N} & 0 & -\sin\phi_{N}\\ 0 & 1 & 0\\ \sin\phi_{N} & 0 & \cos\phi_{N} \end{array}\right]\left[\begin{array}{ccc} 1 & 0 & 0\\ 0 & \cos\phi_{E} & \sin\phi_{E}\\ 0 & -\sin\phi_{E} & \cos\phi_{E} \end{array}\right]\left[\begin{array}{ccc} \cos\phi_{U} & -\sin\phi_{U} & 0\\ \sin\phi_{U} & \cos\phi_{U} & 0\\ 0 & 0 & 1 \end{array}\right]$$

where $\phi ={\left[\phi_{E},\phi_{N},\phi_{U}\right]}^{T}$ denotes the attitude misalignment angles, $\phi_{E}$, $\phi_{N}$, and $\phi_{U}$ correspond to the pitch, roll, and yaw components of the attitude error, respectively.

Since the misalignment angles are sufficiently small in a well-initialized SINS, $C_{n}^{n^{\prime }}$ can be approximated by the first-order Taylor expansion as:

(2) $$C_{n}^{n^{\prime }}\approx I-\left[\phi \times \right]=\left[\begin{array}{ccc} 1 & \phi_{U} & -\phi_{N}\\ -\phi_{U} & 1 & \phi_{E}\\ \phi_{N} & -\phi_{E} & 1 \end{array}\right]$$

where I is the identity matrix, $\left[∙\times \right]$ denotes the skew-symmetric matrix.

### 2.2. Cooperative Navigation Filtering Model

**Figure 1:**
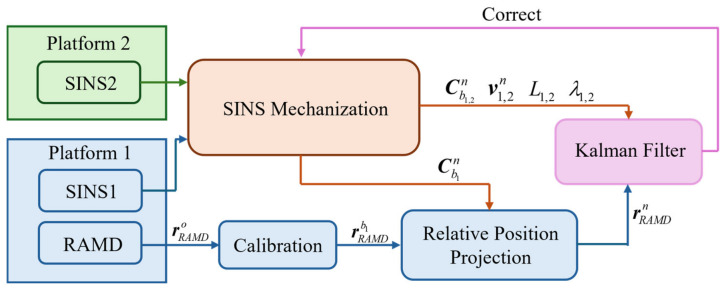
Schematic diagram of dual-platform SINS cooperative navigation.

Based on the data outputs of two SINSs and the relative range and angle measurements between them, the cooperative navigation framework proposed to estimate and compensate cross-platform SINS navigation errors in this manuscript is illustrated in Figure 1.

The RAMD is rigidly mounted on platform 1 together with SINS1, while SINS2 is mounted on platform 2. It measures the relative position of the two SINSs expressed in its own body frame (the o frame), yielding the relative measurement $r_{RAMD}^{o}$. This measurement is then transformed through the calibration matrix $C_{o}^{b_{1}}$ and the attitude matrix $C_{b_{1}}^{n}$ of SINS1 to obtain the relative position of the two SINSs in the n frame, denoted as $r_{RAMD}^{n}$.

The difference between this transformed relative position and the relative position computed by the two SINSs from their navigation solutions is used as the measurement innovation. Finally, a Kalman filter (KF) is employed to estimate the error states, and the attitude, velocity, and position of both SINSs are corrected and output in real time.

#### 2.2.1. State Equation

To establish the state-space model for the KF used in the proposed cooperative navigation framework, the error state equations of the dual-platform system are derived from SINS mechanization theory. The fundamental differential equations of attitude, velocity, and position for a single SINS in the n frame are first given as the physical basis for error analysis.

The attitude update of SINS is governed by the differential equation of the direction cosine matrix:

(3) $${\dot{C}}_{b}^{n}=C_{b}^{n}\left[\omega_{ib}^{b}\times \right]-\left[\omega_{in}^{n}\times \right]C_{b}^{n}$$

where $\omega_{ib}^{b}$ is the angular velocity of the b frame relative to the i frame measured by gyroscopes, $\omega_{in}^{n}=\omega_{ie}^{n}+\omega_{en}^{n}$ denotes the angular velocity of the n frame relative to the i frame, in which $\omega_{ie}^{n}$ is the Earth’s rotational angular velocity, and $\omega_{en}^{n}$ is the angular velocity of the n frame relative to the e frame caused by carrier motion.

The velocity update follows the classical specific force differential equation:

(4) $${\dot{v}}^{n}=C_{b}^{n}f^{b}-\left(2\omega_{ie}^{n}+\omega_{en}^{n}\right)\times v^{n}+g^{n}$$

where $f^{b}$ is the specific force measured by accelerometers, and $g^{n}$ is the local gravity vector.

The position update satisfies the differential relationship between geographic coordinates and velocity:

(5) $$\dot{L}=\frac{1}{R_{N}+h}v_{N},\;\dot{\lambda }=\frac{\sec L}{R_{E}+h}v_{E}$$

where L, λ, and h denote the latitude, longitude and altitude of the carrier, respectively, $v_{E}$, $v_{N}$ are the east and north components of velocity, $R_{N}$ and $R_{E}$ are the meridian radius and prime vertical radius corresponding to the local position, respectively.

Through perturbation analysis and small-angle approximation of attitude misalignment, the error state equations for a single SINS can be derived (detailed derivation steps are provided in Appendix A).

For the dual-SINS cooperative system, the overall error state equation is obtained by combining the error state equations of the two subsystems, as follows:

(6) $${\dot{\phi }}_{1,2}^{n}=-\omega_{in_{1,2}}^{n}\times \phi_{1,2}^{n}+\delta \omega_{in_{1,2}}^{n}-C_{b_{1,2}}^{n}\delta \omega_{ib_{1,2}}^{b_{1,2}}$$

(7) $$\delta {\dot{v}}_{1,2}^{n}=C_{b_{1,2}}^{n}f^{b_{1,2}}\times \phi_{1,2}^{n}-\left(2\omega_{ie_{1,2}}^{n}+\omega_{en_{1,2}}^{n}\right)\times \delta v_{1,2}^{n}-\left(2\delta \omega_{ie_{1,2}}^{n}+\delta \omega_{{\mathrm{en}}_{1,2}}^{n}\right)\times v_{1,2}^{n}+C_{b_{1,2}}^{n}\delta f^{b_{1,2}}$$

(8) $$\delta {\dot{L}}_{1,2}=\frac{1}{R_{N}+h_{1,2}}\delta v_{N_{1,2}},\delta {\dot{\lambda }}_{1,2}=\frac{v_{E_{1,2}}\tan L_{1,2}\sec L_{1,2}}{R_{E}+h_{1,2}}\delta L_{1,2}+\frac{\sec L_{1,2}}{R_{E}+h_{1,2}}\delta v_{E_{1,2}}$$

where $\phi_{1}^{n}$ and $\phi_{2}^{n}$ are the attitude error vectors of the two SINSs, $\delta v_{1}^{n}$ and $\delta v_{2}^{n}$ are the velocity error vectors of the two SINSs, and $\delta L_{1}$, $\delta \lambda_{1}$ and $\delta L_{2}$, $\delta \lambda_{2}$ are the latitude and longitude errors of the two SINSs, respectively. $C_{b_{1}}^{n}$ and $C_{b_{2}}^{n}$ are the attitude rotation matrices from the body frames of the two SINSs to the navigation frame, $f^{b_{1}}$ and $f^{b_{2}}$ are specific forces of the two SINSs, and $h_{1}$ and $h_{2}$ are the altitudes of the two SINSs, respectively.

The total angular velocity error in the navigation frame satisfies $\delta \omega_{in_{1,2}}^{n}=\delta \omega_{ie_{1,2}}^{n}+\delta \omega_{en_{1,2}}^{n}$, which can be explicitly linearized with respect to the position and velocity error components:

(9) $$\delta \omega_{ie_{1,2}}^{n}={\left[\begin{array}{ccc} 0 & -\omega_{ie}\sin L_{1,2}\delta L_{1,2} & \omega_{ie}\cos L_{1,2}\delta L_{1,2} \end{array}\right]}^{T}$$

(10) $$\delta \omega_{en_{1,2}}^{n}={\left[\begin{array}{ccc} \frac{-1}{R_{N}+h_{1,2}}\delta v_{N_{1,2}} & \frac{1}{R_{E}+h_{1,2}}\delta v_{E_{1,2}} & \frac{\tan L_{1,2}}{R_{E}+h_{1,2}}\delta v_{E_{1,2}}+\frac{v_{E_{1,2}}\sec^{2}L}{R_{E}+h_{1,2}}\delta L_{1,2} \end{array}\right]}^{T}$$

$\delta \omega_{ib_{1}}^{b_{1}}$, $\delta \omega_{ib_{2}}^{b_{2}}$ and $\delta f^{b_{2}}$, $\delta f^{b_{2}}$ are the gyro output errors and accelerometer output errors of the two SINSs, respectively, defined as follows:

(11) $$\begin{array}{l} \delta \omega_{ib_{1,2}}^{b_{1,2}}=\varepsilon^{b_{1,2}}+w_{g_{1,2}},{\dot{\varepsilon }}^{b_{1,2}}=0_{3\times 1},w_{g_{1,2}}∼N\left(0,Q_{g_{1,2}}\right)\\ \delta f^{b_{1,2}}=\nabla^{b_{1,2}}+w_{a_{1,2}},{\dot{\nabla }}^{b_{1,2}}=0_{2\times 1},w_{a_{1,2}}∼N\left(0,Q_{a_{1,2}}\right) \end{array}$$

where $\varepsilon^{b_{1}}$ and $\varepsilon^{b_{2}}$ are the gyro biases of the two SINSs, $w_{g_{1}}$ and $w_{g_{1}}$ are the corresponding gyro noises. $\nabla^{b_{1}}$ and $\nabla^{b_{2}}$ are the accelerometer bias errors of the two SINSs, $w_{a_{1}}$ and $w_{a_{2}}$ are the corresponding accelerometer random walk errors. $Q_{g_{1}}$, $Q_{g_{2}}$ and $Q_{a_{1}}$, $Q_{a_{2}}$ are the covariance matrices of the gyro and accelerometer random noises for the two SINSs, respectively.

It should be noted that since the coupling between the vertical channel and the horizontal channels is weak and the vertical channel can be damped, this paper does not consider vertical-related errors, including vertical velocity error, position error, and accelerometer bias.

The error state vector of the cooperative navigation system is as follows:

(12) $$X\left(t\right)=\begin{array}{cccccccccccc} \left[\delta v_{E_{1}}\right. & \delta v_{N_{1}} & \phi_{E_{1}} & \phi_{N_{1}} & \phi_{U_{1}} & \delta L_{1} & \delta \lambda_{1} & \varepsilon_{x}^{b_{1}} & \varepsilon_{y}^{b_{1}} & \varepsilon_{z}^{b_{1}} & \nabla_{x}^{b_{1}} & \nabla_{y}^{b_{1}}\\ \delta v_{E_{2}} & \delta v_{N_{2}} & \phi_{E_{2}} & \phi_{N_{2}} & \phi_{U_{2}} & \delta L_{2} & \delta \lambda_{2} & \varepsilon_{x}^{b_{2}} & \varepsilon_{y}^{b_{2}} & \varepsilon_{z}^{b_{2}} & \nabla_{x}^{b_{2}} & \left.\nabla_{y}^{b_{2}}\right] \end{array}$$

The state equation of the cooperative navigation system is given by:

(13) $$\dot{X}\left(t\right)=F\left(t\right)X\left(t\right)+G\left(t\right)W\left(t\right)$$

where $F\left(t\right)$ is the state transition matrix of the cooperative navigation system, which is constructed from the aforementioned error state equations (Equations (6)–(8)). Since the error propagation of the two SINS subsystems is independent of each other, $F\left(t\right)$ takes a block-diagonal form: $F\left(t\right)=\left[\begin{array}{cc} F_{1}\left(t\right) & 0_{12\times 12}\\ 0_{12\times 12} & F_{2}\left(t\right) \end{array}\right]$, in which the two submatrices share the same structure and are uniformly denoted as:

(14) $$F_{1,2}\left(t\right)=\left[\begin{array}{ccccc} -J\left[\left(2\omega_{ie_{1,2}}^{n}+\omega_{en_{1,2}}^{n}\right)\times \right]J^{T} & \left[{\bar{C}}_{b_{1,2}}^{n}f^{b_{1,2}}\times \right] & M_{13} & 0_{2\times 2} & {\bar{C}}_{b_{1,2}}^{n}\\ M_{21} & -\omega_{in_{1,2}}^{n} & M_{23} & -C_{b_{1,2}}^{n} & 0_{3\times 3}\\ M_{31} & 0_{2\times 3} & M_{33} & 0_{2\times 3} & 0_{2\times 2}\\ 0_{3\times 2} & 0_{3\times 3} & 0_{3\times 2} & 0_{3\times 3} & 0_{3\times 2}\\ 0_{2\times 2} & 0_{2\times 3} & 0_{2\times 2} & 0_{2\times 3} & 0_{2\times 2} \end{array}\right]$$

$W\left(t\right)$ is the process noise vector, and the corresponding noise driving matrix $G\left(t\right)$ is expressed as:

(15) $$G\left(t\right)=\left[\begin{array}{cccc} 0_{2\times 3} & {\bar{C}}_{b_{1}}^{n} & 0_{2\times 3} & 0_{2\times 2}\\ -C_{b_{1}}^{n} & 0_{3\times 2} & 0_{3\times 3} & 0_{3\times 2}\\ 0_{7\times 3} & 0_{7\times 2} & 0_{7\times 3} & 0_{7\times 2}\\ 0_{2\times 3} & 0_{2\times 2} & 0_{2\times 3} & {\bar{C}}_{b_{2}}^{n}\\ 0_{3\times 3} & 0_{2\times 2} & -C_{b_{2}}^{n} & 0_{2\times 2}\\ 0_{7\times 3} & 0_{7\times 2} & 0_{7\times 3} & 0_{7\times 2} \end{array}\right]$$

where $\begin{array}{l} {\bar{C}}_{b_{1}}^{n}=JC_{b_{1}}^{n}J^{T}\\ {\bar{C}}_{b_{2}}^{n}=JC_{b_{2}}^{n}J^{T} \end{array}$, $J=\left[\begin{array}{ccc} 1 & 0 & 0\\ 0 & 1 & 0 \end{array}\right]$, the parameter matrix M in $F_{1}\left(t\right)$, and $F_{2}\left(t\right)$ can be found in Appendix B.

#### 2.2.2. Observation Equation

**Figure 2:**
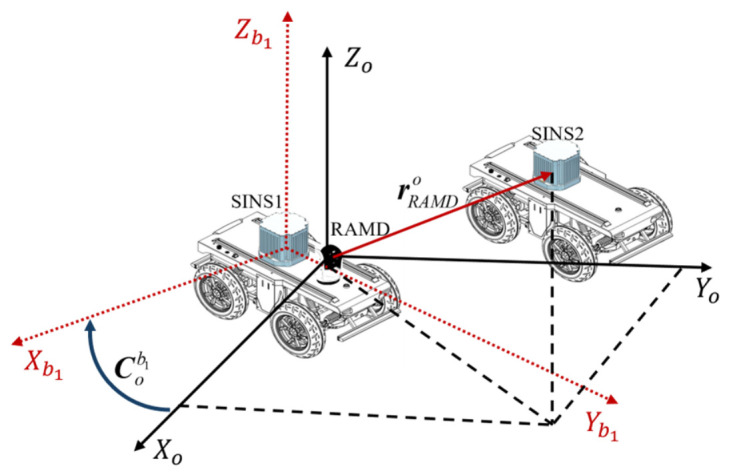
Schematic diagram of relative ranging and angle measurement.

A schematic diagram of the relative ranging and angle measurement is shown in Figure 2.

After high-accuracy pre-calibration, the calibration matrix $C_{o}^{b_{1}}$ between RAMD and SINS1 can be obtained. The relative position between the two SINSs measured by RAMD and projected in the n frame is then given by:

(16) $${\tilde{r}}_{RAMD}^{n}=C_{b_{1}}^{n^{\prime }}C_{o}^{b_{1}}{\tilde{r}}_{RAMD}^{o}=\left(I-\left[\phi_{1}^{n}\times \right]\right)C_{b_{1}}^{n}C_{o}^{b_{1}}\left(r_{RAMD}^{o}+\delta r_{RAMD}^{o}\right)$$

On the other hand, the cooperative navigation algorithm outputs the absolute position estimates of the two SINSs in the n frame. The relationship among true positions, estimated positions, and position estimation errors is defined as:

(17) $$\left\{\begin{array}{c} {\hat{R}}_{SINS1}^{n}=R_{SINS1}^{n}+\delta R_{SINS1}^{n},\delta R_{SINS1}^{n}={\left[\begin{array}{cc} \delta L_{1} & \delta \lambda_{1} \end{array}\right]}^{T}\\ {\hat{R}}_{SINS2}^{n}=R_{SINS2}^{n}+\delta R_{SINS2}^{n},\delta R_{SINS2}^{n}={\left[\begin{array}{cc} \delta L_{2} & \delta \lambda_{2} \end{array}\right]}^{T} \end{array}\right.$$

where $R_{SINS1}^{n}$ and $R_{SINS2}^{n}$ are the true position vectors of SINS1 and SINS2, respectively, while $\delta R_{SINS1}^{n}$ and $\delta R_{SINS2}^{n}$ are the corresponding estimation errors.

Based on Equation (17), the estimated relative position vector between the two SINSs in the n frame can be further derived as:

(18) $${\hat{r}}_{SINS}^{n}=U\left({\hat{R}}_{SINS2}^{n}-{\hat{R}}_{SINS1}^{n}\right)=U\left[\left(R_{SINS2}^{n}-R_{SINS1}^{n}\right)-\left(\delta R_{SINS2}^{n}-\delta R_{SINS1}^{n}\right)\right]$$

where U denotes the position scaling matrix, which converts the latitude and longitude differences of the two SINSs into the horizontal relative position vector (in meters) in the navigation frame, expressed as $U=\left[\begin{array}{cc} R_{N}+h_{1} & 0\\ 0 & \left(R_{E}+h_{1}\right)\cos L_{1} \end{array}\right]$.

By subtracting Equation (18) from Equation (16) and neglecting second-order small error terms, the linearized observation model of the cooperative navigation system is obtained as Equation (19), which characterizes the error relationship between the RAMD measurement and the relative position estimates from the cooperative navigation solution.

(19) $$Z_{SINS/RAMD}={\tilde{r}}_{RAMD}^{n}-{\hat{r}}_{SINS}^{n}\approx r_{RAMD}^{n}\times \phi_{1}^{n}+U\left(\delta R_{SINS2}^{n}-\delta R_{SINS1}^{n}\right)+\delta r_{RAMD}^{n}$$

In summary, the observation equation and observation matrix of the cooperative navigation system are given as follows:

(20) $$Z\left(t\right)=H\left(t\right)X\left(t\right)+V\left(t\right)$$

(21) $$H\left(t\right)=\left[\begin{array}{cccccc} 0_{2\times 2} & \left[r_{RAMD}^{n}\times \right] & -U & 0_{2\times 10} & U & 0_{2\times 5} \end{array}\right]$$

where $H\left(t\right)$ denotes the observation matrix, and $V\left(t\right)$ corresponds to the RAMD measurement error $\delta r_{RAMD}^{n}$, which is modeled as Gaussian white noise.

## 3. Observability and Estimability Analysis

### 3.1. Observability and Estimability Analysis Method

By using FIM, this paper quantitatively analyzes how different maneuver types affect the observability of a cooperative navigation system. While the method in [28] applies SVD to the FIM and builds a state correlation matrix to examine dependencies among observable states, thereby revealing which states are coupled, it does not identify which linear combinations of states are fundamentally unobservable, nor does it prescribe how to break those couplings through motion design. By contrast, this work directly extracts the unobservable linear combinations of states from the FIM null space. Building upon these unobservable combinations revealed under specific motions, the proposed analysis establishes a direct link between observability characteristics and trajectory design, thereby providing explicit motion design principles for improving the observability of the cooperative navigation system.

Discretizing the state space of the cooperative navigation system consisting of Equations (13) and (20) yields:

(22) $$\begin{array}{l} x_{k+1}=\Phi_{k}x_{k}+w_{k},w_{k}∼Ν\left(0,Q_{k}\right)\\ z_{k}=H_{k}x_{k}+v_{k},v_{k}∼Ν\left(0,R_{k}\right) \end{array}$$

where $Q_{k}$ is the process noise covariance matrix of the system, and $R_{k}$ is the measurement noise covariance matrix. The information matrix $L_{k}$ can be expressed as:

$${\bar{\Phi }}_{k}=\prod_{n=k}^{1}\Phi_{n}=\Phi_{n}\cdot \Phi_{n-1}\cdot \ldots \cdot \Phi_{1}$$

(23) $$L_{k}=\sum_{n=1}^{k}{\bar{\Phi }}_{n}^{T}\cdot H_{n}^{T}\cdot R_{n}^{-1}\cdot H_{n}\cdot {\bar{\Phi }}_{n}$$

After performing SVD on the information matrix $L_{k}$, if a singular value $\sigma_{i}$ exceed $\sigma_{\max}\times 10^{-16}$ and the corresponding singular vector elements $u_{i}$ satisfy $\left|u_{i}\right|>0.1$, the associated state combination is deemed observable, which implies that sufficient information can be extracted from the measurements to distinguish it from numerical roundoff errors.

In general, initial state uncertainty, process noise, and measurement noise all affect state estimation errors. The PWCS method extracts observability matrices in time segments and computes eigenvalues and eigenvectors via SVD to analyze the degree of observability. However, its results are not sufficiently accurate because it does not account for the influence of random noise [29]. The covariance analysis method evaluates state estimability by analyzing the eigenvalues and eigenvectors of the KF estimation error covariance matrix. Although it considers the effects of initial state covariance, process noise, and measurement noise matrices, yielding more scientifically sound results, it is a posteriori method, and its conclusions are heavily influenced by the initial error covariance settings [30].

The FIM approach reflects the inherent properties of the system model. It only considers the measurement noise covariance and does not depend on the initial covariance setting, thus providing a more objective assessment of the system’s intrinsic observability [31]. In this paper, the condition number κ of FIM is used to quantitatively evaluate system observability [32], defined as

(24) $$\kappa \left(L_{k}\right)=\frac{\lambda_{\max}\left(L_{k}\right)}{\lambda_{\min}\left(L_{k}\right)}$$

where $\lambda_{\max}\left(L_{k}\right)$ and $\lambda_{\min}\left(L_{k}\right)$ represent the maximum and minimum nonzero eigenvalues of the FIM, respectively. A smaller condition number implies more uniform distribution of information over all state dimensions and superior system observability. By contrast, an excessively large or infinite condition number corresponds to an ill-conditioned system containing unobservable state directions. This criterion is adopted throughout the present work to quantitatively evaluate system observability under diverse maneuver conditions.

### 3.2. Observability Analysis of the Cooperative Navigation System Under Different Maneuvers

This paper investigates how various maneuver conditions affect the observability of a dual-SINS cooperative navigation system constrained by relative range and angle measurements. The FIM condition number is adopted to quantitatively evaluate state estimability under typical motion scenarios. Combined with the right null-space decomposition of the FIM, unobservable state combinations corresponding to each working condition are determined. On this basis, a concise planar trajectory is designed to realize full-bias estimation and suppress yaw as well as positioning errors of each SINS. Notably, only planar movements including straight-line acceleration/deceleration and turning are required, and pitch/roll-axis excitation is unnecessary. This feature fits the practical characteristics of unmanned ground vehicles (UGVs), which are incapable of persistent pitch and roll maneuvers as unmanned aerial vehicles (UAVs) do.

**Figure 3:**
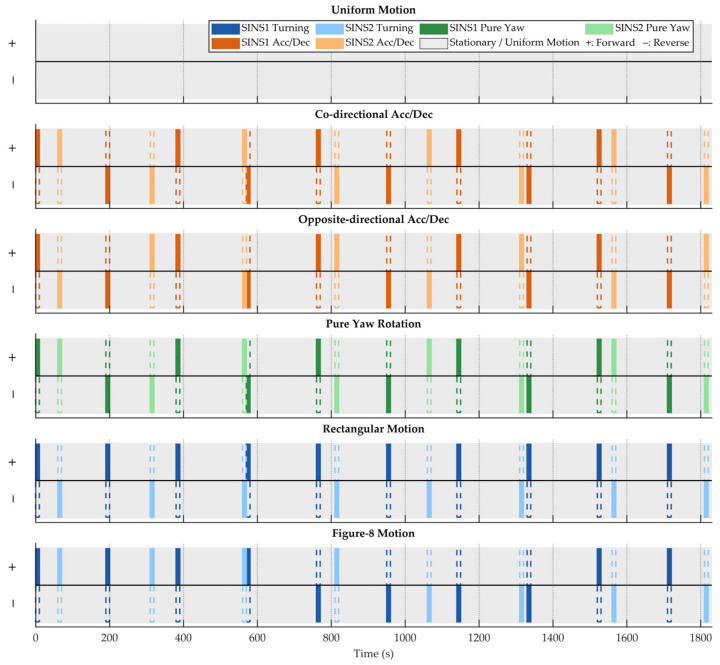
Six typical motion schemes examined.

This paper compares six typical motion schemes: co-directional longitudinal acceleration/deceleration, opposite-direction longitudinal acceleration/deceleration, pure yaw rotation, rectangular motion, figure-8 motion and uniform motion. To guarantee the validity of comparative analysis, identical acceleration and angular velocity magnitudes are specified for all acceleration, deceleration and turning segments, as illustrated in Figure 3. Colored bars distinguish motion types (blue: turning, green: pure yaw rotation, orange: acceleration/deceleration), while gray areas represent stationary or uniform motion. The filled segments of the bars indicate movement directions, where the symbols “+” and “−” on the left represent forward and reverse directions, respectively. Correspondingly, the acceleration and deceleration magnitudes of the two SINS are preset to $1.2\;m/s^{2}$ and $2.4\;m/s^{2}$, respectively, the angular velocity amplitude is set to 22.5°/s. The stationary and uniform-motion durations of SINS1 and SINS2 are set to 180 s and 240 s, respectively, and the measurement noise is configured as $\left[1,1,0.5\right]\;m^{2}$.

It is worth noting that stationary or uniform-motion periods themselves do not contribute observable information, but appropriate stationary/uniform-motion intervals help reduce numerical errors and promote filter convergence [33]. In this paper, different stationary/uniform-motion durations are set for the two SINSs to achieve asynchronous alternating motion. Meanwhile, repetitive periodic motion of the two SINSs is performed, which helps maintain a balanced information contribution from different time windows, thereby enhancing the numerical stability of the FIM calculation.

**Figure 4:**
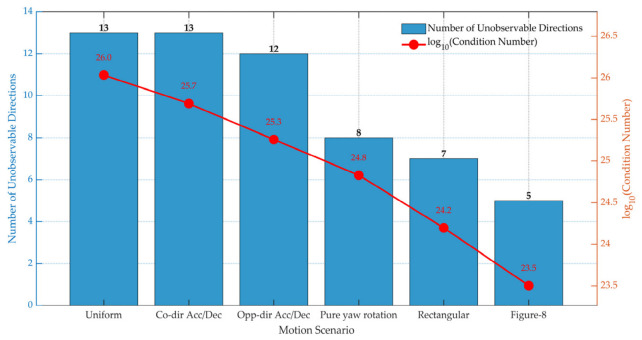
Comparison of system observability across different motions.

Figure 4 shows the number of null-space unobservable directions and the FIM condition number under different maneuvers. Compared with uniform motion, co-directional Acc/Dec neither reduces the number of unobservable directions nor significantly decreases the condition number, indicating that longitudinal acceleration/deceleration without yaw change contributes little to system observability—a conclusion consistent with [16,23]. From the bars for co-directional Acc/Dec, opposite-directional Acc/Dec, and pure yaw rotation, it is observed that as the two SINSs undergo more diverse yaw angle changes, the number of unobservable directions drops rapidly, and the condition number decreases accordingly. This suggests that yaw angle variation is the dominant factor in improving system observability. Nevertheless, relying solely on pure yaw rotation still yields limited observability enhancement.

Compared with pure yaw rotation, both rectangular and figure-8 motions involve turns that simultaneously change the yaw angle and induce acceleration variations, thereby helping to decouple the unobservable linear combinations of states. As shown in Figure 4, the introduction of turning maneuvers reduces the FIM condition number by one order of magnitude relative to pure yaw rotation, and by two orders of magnitude relative to pure longitudinal acceleration/deceleration, the number of unobservable directions also drops significantly. These results indicate that, for a dual-SINS cooperative navigation system constrained by relative range and angle measurements, even a simple planar trajectory can substantially enhance observability and thus reduce positioning errors.

**Figure 5:**
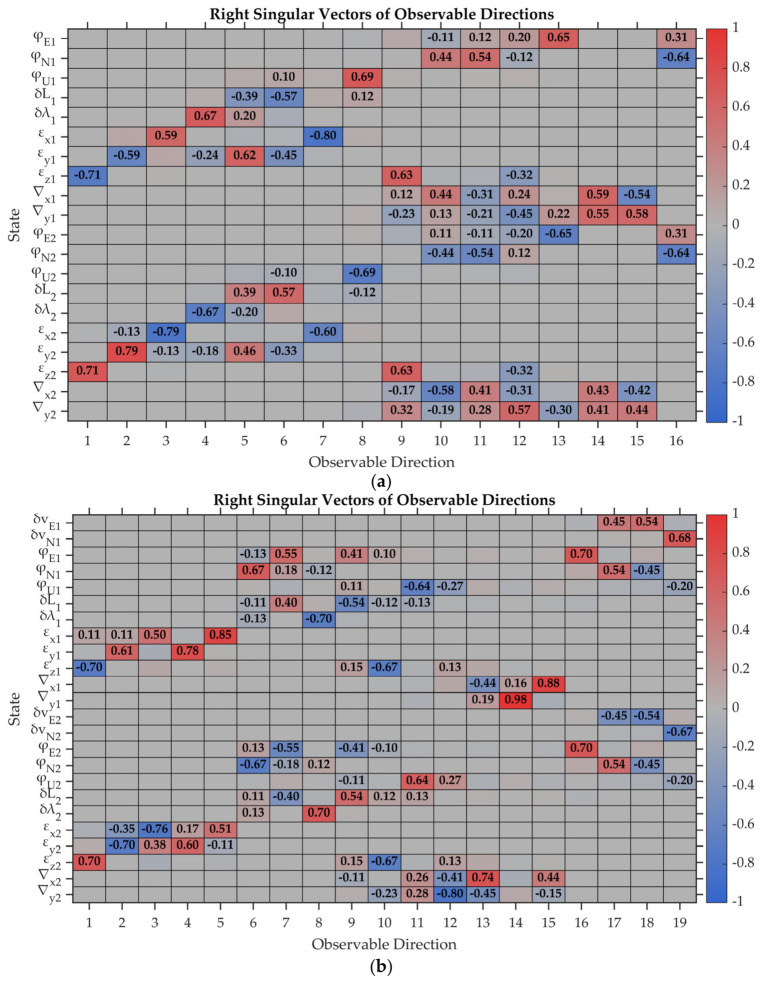
Singular vector coefficients for unobservable directions: (**a**) pure yaw rotation; (**b**) figure-8 Motion.

To further investigate the observability improvement from turning maneuvers compared with pure yaw rotation, the right singular vectors corresponding to the observable directions under several typical motions are extracted, and the significant components with absolute values greater than a threshold of 0.1 are visualized, as shown in Figure 5. In the figure, the horizontal axis represents the observable direction index (ranked by descending singular values), and the vertical axis lists the state variables that appear in these observable directions. The color intensity of each cell indicates the magnitude and sign of the corresponding coefficient in the singular vector (blue for negative, red for positive, and gray for coefficients close to zero), while the numeric value inside the cell shows the specific coefficient (displayed only when its absolute value is ≥0.1). This figure intuitively illustrates which linear combinations of states dominate each observable direction.

It should be noted that observable directions only indicate that a combination of state variables is observable, and do not imply that every involved state variable is observable individually. In Figure 5, clear linear dependencies emerge among the state variables, as the relative position difference between the two SINSs acts as the measurement constraint. For example, in the first column of Figure 5a, the differential mode of the z-axis gyro biases of the two SINSs ($\left|\varepsilon_{z_{1}}-\varepsilon_{z_{2}}\right|$) constitutes an observable direction, which reflects the inherent observability of the system determined jointly by the state equation and the observation equation. To further separate the gyro biases of the two SINSs into individually observable states (i.e., to make the common-mode $\left|\varepsilon_{z_{1}}+\varepsilon_{z_{2}}\right|$ observable as well), additional motion excitation is required. In general, the fewer the state variables involved in an observable direction and the larger the absolute values of their coefficients, the higher the estimability of the individual states.

Figure 5b shows that the common-mode of the two SINSs ($\left|\delta v_{E_{1}}+\delta v_{E_{2}}\right|$, $\left|\delta v_{E_{1}}+\delta v_{E_{2}}\right|$,$\left|\delta L_{1}+\delta L_{2}\right|$, $\left|\delta \lambda_{1}+\delta \lambda_{2}\right|$) does not appear in the observable directions, indicating that under the constraints of relative range and angle measurements and motion excitation, the state variables of a single SINS ($\delta v_{E}$, $\delta v_{N}$, δL, δλ) remain unobservable. However, by designing a proper motion trajectory (making the biases of each axis, especially the z-axis gyro bias, observable), the negative impact of these unobservable common-modes can be effectively suppressed, thereby improving the absolute positioning accuracy.

Under the figure-8 motion, the coefficients of the common-mode gyro bias combinations ($\left|\varepsilon_{x_{1}}+\varepsilon_{x_{2}}\right|$, $\left|\varepsilon_{y_{1}}+\varepsilon_{y_{2}}\right|$, $\left|\varepsilon_{z_{1}}+\varepsilon_{z_{2}}\right|$) are concentrated in directions 4, 5, and 10, respectively, with little coupling to other state variables. This indicates that these common-mode components can be clearly separated, and the observability of the x- and y-axis gyro biases is higher than that of the z-axis. In contrast, in Figure 5a, only the x-axis gyro bias common-mode ($\left|\varepsilon_{x_{1}}+\varepsilon_{x_{2}}\right|$) has relatively concentrated coefficient distribution, while the y- and z- axis gyro bias common-modes ($\left|\varepsilon_{y_{1}}+\varepsilon_{y_{2}}\right|$, $\left|\varepsilon_{z_{1}}+\varepsilon_{z_{2}}\right|$) have coefficients distributed across multiple directions and are coupled with other state variables, making them difficult to separate and resulting in low observability. In Figure 5b, the coefficients of $\nabla_{y_{2}}$, $\nabla_{x_{2}}$, $\nabla_{y_{1}}$, $\nabla_{x_{1}}$ in directions 12, 13, 14, 15, respectively, exceed 0.7, indicating that the x- and y-axis accelerometer biases are effectively decoupled with high observability. Compared with Figure 5b, the coefficient distribution of the accelerometer common-mode in Figure 5a is significantly more dispersed, and the corresponding observable directions involve multiple state variables, leading to relatively poor observability. The above analysis shows that turning motions (which simultaneously change the yaw angle and horizontal acceleration) can effectively improve the observability of gyro biases and accelerometer biases in the cooperative navigation system compared with pure yaw rotation, thereby enhancing the absolute positioning accuracy.

Moreover, Figure 5b exhibits observable directions (directions 16–19) for the attitude error common-mode ($\left|\phi_{E_{1}}+\phi_{E_{2}}\right|$, $\left|\phi_{N_{1}}+\phi_{N_{2}}\right|$, $\left|\phi_{U_{1}}+\phi_{U_{2}}\right|$) that are absent in Figure 5a. This indicates that turning motions can improve the observability of the attitude error of each single SINS, further enhancing the absolute positioning accuracy.

In summary, under the constraints of relative range and angle measurements, by maneuvering the two SINSs to perform relative turning motions in the horizontal plane—thereby inducing simultaneous variations in both yaw angle and horizontal acceleration—and by inserting appropriate stationary or constant-velocity intervals to facilitate filter convergence, the observability of the biases on each axis can be effectively improved, which in turn enhances the positioning accuracy of the SINSs employed. To validate this conclusion, a simulation experiment will be conducted in the following section.

## 4. Simulation

Based on the observability analysis, a simulation is conducted to analyze the bias estimation and absolute positioning accuracy improvement of two SINSs performing a figure-8 motion under the constraints of relative range and angle measurements. A sliding-window FIM analysis is also conducted to intuitively demonstrate the observability improvement brought by turning maneuvers. It should be noted that the figure-8 motion is one of the relatively simple trajectories proposed in this paper that enables estimation of all biases.

The error parameters of the two SINSs configured in the simulation are listed in Table 2.

**Figure 6:**
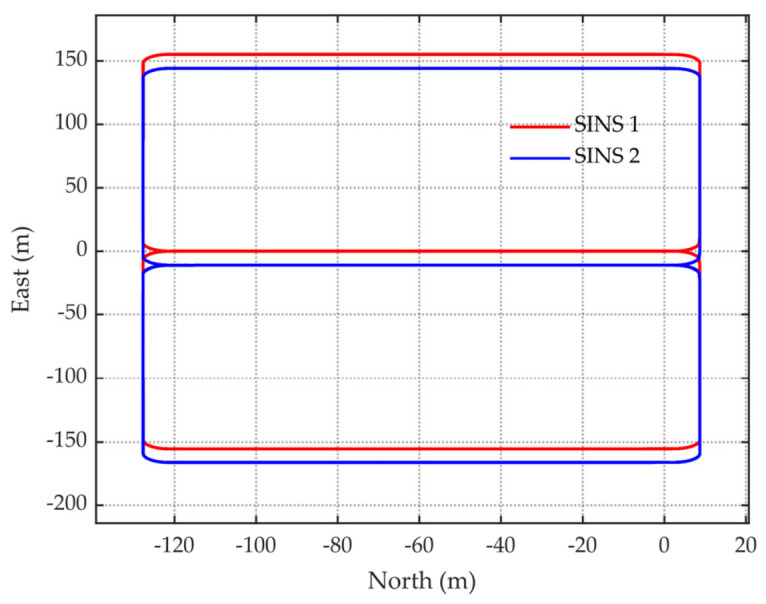
Simulation results: trajectories of the two SINSs.

In the initial stage of the simulation, the distance between the two platforms is set as 20 m, with SINS1 mounted on platform 1 and SINS2 on platform 2. A 4 h of cooperative navigation is performed: for the first 3.5 h, the two SINSs move along the figure-8 trajectory as shown in Figure 3, for the remaining 0.5 h, they stay stationary. The motion trajectories of the two SINSs are shown in Figure 6.

**Figure 7:**
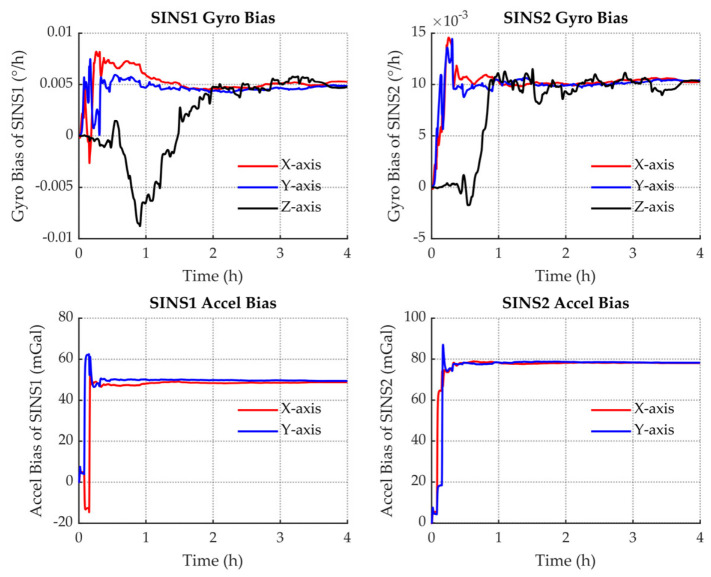
Simulation results: bias estimation results of the two SINSs.

**Figure 8:**
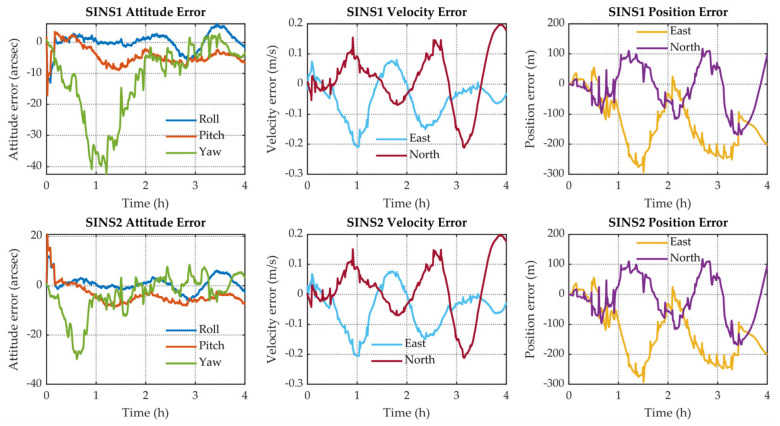
Simulation results: navigation errors of the two SINSs.

The bias estimation results of the cooperative navigation system are shown in Figure 7, and the attitude, velocity, and position error curves are provided in Figure 8. As indicated in Figure 7, constrained by relative range and angle measurements, the figure-8 motion enables rapid convergence of the x- and y-axis gyro and accelerometer biases for both SINSs, with the estimates approaching the true values within 1 h. The z-axis gyro bias converges in approximately 2 h, matching the preceding observability analysis. Once the navigation filter converges, the steady-state estimation errors are below 0.001°/h for the x- and y-axis gyro biases, below 0.002°/h for the z-axis gyro bias, and below 2 mGal for the x- and y-axis accelerometer biases of the two SINSs.

Figure 8 shows that although the velocity, position, and z-axis attitude errors of a single SINS are theoretically unobservable, cooperative navigation still effectively suppresses the divergence of these errors. With the convergence of the x- and y-axis gyro biases and the z-axis gyro bias, the divergence of the attitude errors (especially the z-axis attitude error) of the two SINSs is effectively suppressed. Meanwhile, the convergence of the x- and y-axis accelerometer biases also helps to suppress the divergence of velocity errors. After 4 h of cooperative navigation, the east and north position errors of each single SINS do not exceed 300 m, and the positioning accuracy is significantly improved compared with single SINS navigation result.

Furthermore, the velocity and position errors of the two SINSs exhibit almost identical trends over time, which is also consistent with the observability analysis conclusion: under only relative range and angle measurement constraints, the two-dimensional planar motion combination of the two SINSs is insufficient to make the common-mode errors ($\left|\delta v_{E_{1}}+\delta v_{E_{2}}\right|$, $\left|\delta v_{E_{1}}+\delta v_{E_{2}}\right|$, $\left|\delta L_{1}+\delta L_{2}\right|$, $\left|\delta \lambda_{1}+\delta \lambda_{2}\right|$) observable. More specifically, relative position measurements act as direct observations for the cooperative Kalman filter, which accurately estimates and suppresses the inter-system relative position errors. Owing to the inherent derivative relationship between position and velocity in inertial error propagation, the convergence of relative position errors inherently drives the relative velocity errors to converge synchronously, despite relative velocity not being directly observed. As both relative position and velocity errors converge to near zero, the mismatch between the absolute error profiles of the two SINSs is correspondingly minimized, which explains the highly consistent trajectories of the two sets of error curves.

**Figure 9:**
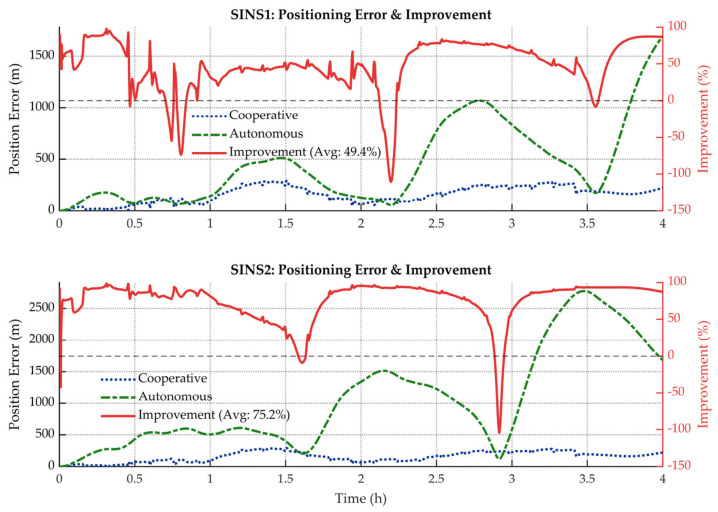
Simulation results: positioning errors of autonomous and cooperative navigation modes.

Figure 9 compares the positioning errors of SINS1 and SINS2 under autonomous and cooperative navigation modes. The red curves represent the accuracy improvement rate η of cooperative navigation over autonomous navigation, defined as:

(25) $$\eta =\frac{\delta R_{\mathrm{auto}}-\delta R_{\mathrm{coop}}}{\delta R_{\mathrm{auto}}}\times 100\%$$

where $\delta R_{\mathrm{auto}}$ and $\delta R_{\mathrm{coop}}$ denote the positioning errors under the two modes, respectively. After 1 h, the improvement rate curves of both SINSs generally follow the same trend as the autonomous navigation error curves, indicating that once the bias estimates converge, the autonomous navigation errors continue to diverge over time, making the improvement brought by cooperative navigation increasingly significant. Over the 4 h simulation, the average positioning accuracy improvement is 49.4% for SINS1 and 75.2% for SINS2. The larger improvement in SINS2 is due to the fact that it was assigned a lower-accuracy sensor in the simulation, resulting in larger autonomous navigation errors and thus a more pronounced improvement. This suggests that the proposed method achieves greater improvement when applied to lower-precision SINSs.

**Figure 10:**
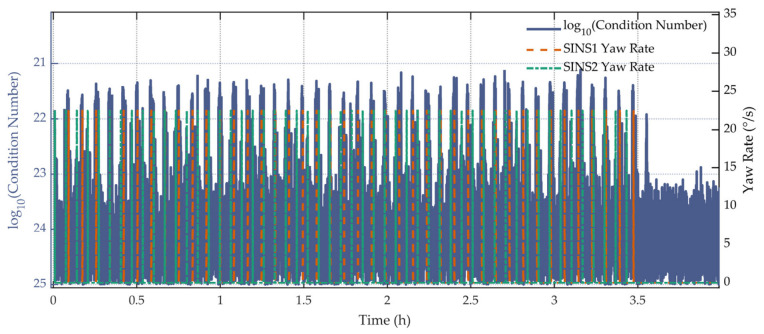
Simulation results: effect of turning maneuvers on system observability.

A sliding-window FIM analysis with a window size of 30 s is performed to further quantify the effect of turning maneuvers on system observability. The condition number calculation results are presented in Figure 10, where the left y-axis shows the condition number on a logarithmic scale and the right y-axis plots the angular velocity of SINS1 and SINS2. The time intervals in which the condition number drops coincide closely with the intervals of elevated angular velocity. Each turning maneuver significantly reduces the condition number, and the values within windows that contain turning maneuvers are approximately two orders of magnitude lower than those in uniform-motion or stationary segments. This contrast directly indicates that such maneuvers can improve system observability by roughly two orders of magnitude.

The simulation results presented in this section validate the proposed observability analysis through bias estimation and sliding-window FIM verification. The results confirm that cooperative navigation along a figure-8 trajectory enables full estimation of all inertial sensor biases and effectively suppresses navigation error divergence. To further validate the effectiveness of the proposed method, field experiments are carried out in the next section.

## 5. Field Experiments

Field experiments are carried out using two SINSs, one of which is an RLG-50-INS (SINS1), while the other is an RLG-90-INS (SINS2), both developed by National University of Defense Technology, Changsha, China. Since no independent relative ranging and angle measurement device was available during the tests, the relative range and angle information is obtained by GNSS inversion with simulated noise [34]. The noise parameters are set according to the nominal accuracy of the RH-6 electro-optical pod (manufactured by Ruihang, Beijing, China), and the corresponding device parameters are listed in Table 3.

During the experiments, SINS1 and SINS2 are mounted on different UGVs. A GNSS receiver is mounted next to each SINS to provide positioning information. All raw data undergo time synchronization and lever-arm compensation prior to processing.

On each individual platform, the SINS and its paired GNSS receiver share a unified hardware clock, ensuring native time alignment between inertial measurements and GNSS outputs. Offline inter-platform time alignment is further performed based on the GNSS pulse-per-second (PPS) signals from both receivers, with the inter-system synchronization error controlled at the millisecond level. Given the low dynamics of the UGVs, such millisecond-level timing errors translate into minimal position offsets and have virtually no impact on the experimental results.

For lever-arm compensation, the 3D lever-arm vector of each GNSS antenna relative to the measurement center of its corresponding SINS is calibrated prior to field tests, and the corresponding lever-arm error is compensated in the coordinate transformation procedure. The residual error after compensation is far below the noise level of the relative range and angle measurements, and thus has negligible impact on the final navigation accuracy.

**Figure 11:**
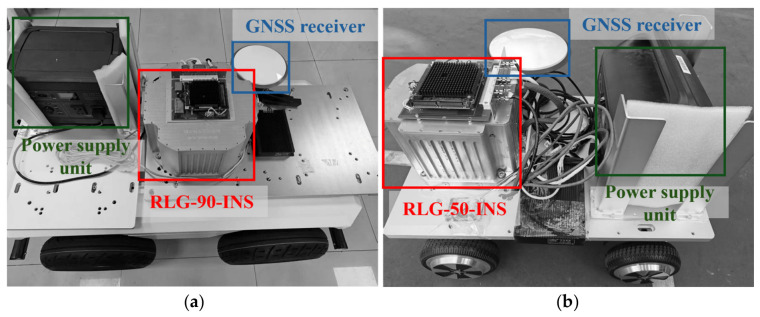
Actual vehicle test platform: (**a**) RLG-90-INS, (**b**) RLG-50-INS.

**Figure 12:**
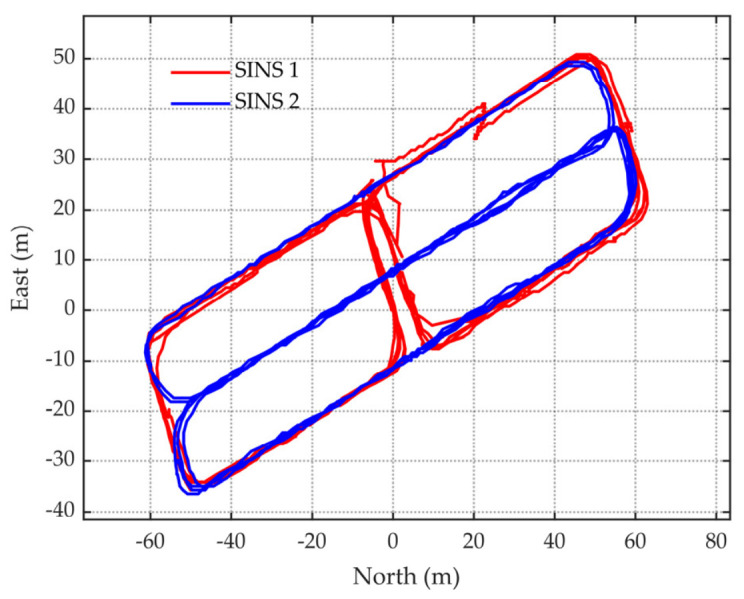
Field experiment results: trajectories of the two SINSs.

Figure 11 shows the two UGV test platforms used in the field experiments. The total experiment duration was 4 h. SINS1 and SINS2 remained stationary for 1 h to complete GNSS-aided initial alignment, after which they operated in cooperative navigation mode for 3 h, aided by relative range and angle measurements. During the experiment, both UGVs traveled along a figure-8 trajectory around a basketball court, matching the simulated trajectory. The reference trajectories from the GNSS receivers are shown in Figure 12.

Since no dedicated relative ranging and angle measurement device was available during the field tests, we followed a strategy similar to that reported in [34], which is commonly used for algorithm validation in such scenarios. The specific implementation steps are as follows:

Step 1: Based on the raw GNSS data collected from both platforms, the high-accuracy relative position between the two SINSs in the navigation frame was calculated using the GNSS carrier-phase differential technique. This result was taken as the noise-free reference for the subsequent simulation.

Step 2: This reference relative position was projected into the body frame of SINS1 using the high-accuracy attitude matrix from the GNSS/SINS integrated navigation solution, yielding the ideal relative range and yaw-angle measurements that would be provided by a noise-free electro-optical pod.

Step 3: According to the performance specifications of the RH-6 electro-optical pod (see Table 3), Gaussian white noise with a standard deviation of 1% of the ideal range was added to the range measurement, and Gaussian white noise with a standard deviation of $1^{\prime }$ was added to the yaw-angle measurement.

Step 4: The noise-contaminated range and angle measurements were projected back into the navigation frame using the real-time attitude matrix from the cooperative navigation solution of SINS1, thereby generating the observations actually fed into the cooperative navigation filter.

**Figure 13:**
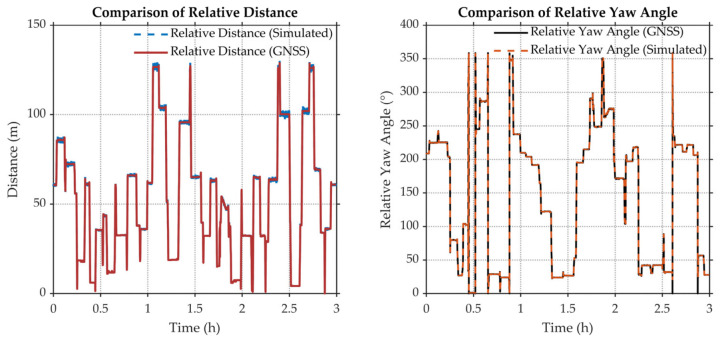
Relative distance and yaw angle: comparison between simulated results and GNSS reference.

Figure 13 compares the simulated relative range and yaw angle with the reference values derived from GNSS.

**Figure 14:**
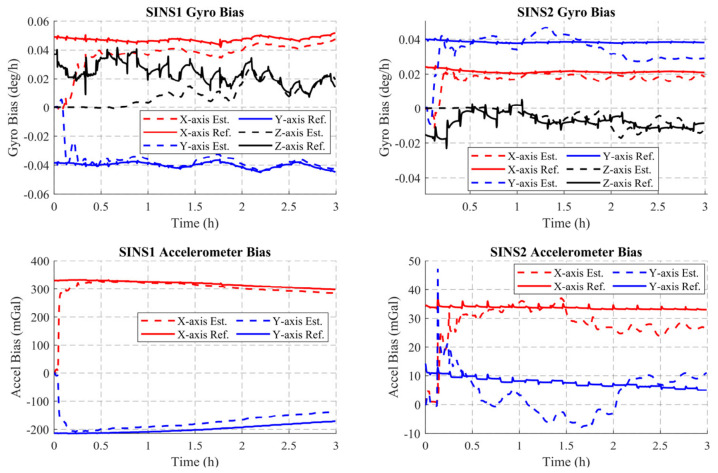
Field experiment results: bias estimation results of the two SINSs.

**Figure 15:**
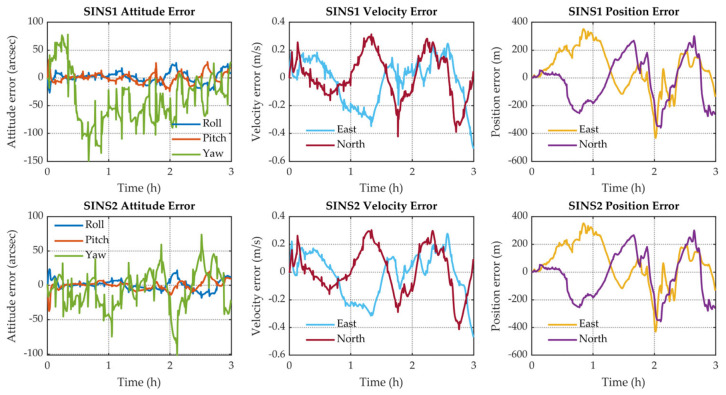
Field experiment results: navigation errors of the two SINSs.

Figure 14 and Figure 15 present the bias estimation results and the navigation error results, respectively. In Figure 14, the gyroscope and accelerometer biases estimated by GNSS/INS integrated navigation are used as a reference [35]. It can be seen that as the UGVs repeatedly undergo turning maneuvers along the prescribed trajectory, with continuously varying acceleration and angular velocity, the biases of each axis of the two SINSs gradually become observable, and the estimated biases approach the reference values.

To further quantify the benefit of the bias estimation, an improvement metric is defined for each SINS axis as:

(26) $$I_{i}=\left(1-\frac{R_{i}}{\left|{\bar{b}}_{ref}\right|}\right)\times 100\%$$

where $R_{i}$ denotes the RMSE of the estimated bias with respect to the reference after filter convergence, and ${\bar{b}}_{reF}$ is the mean of the reference bias sequence for that axis over the same period. The index i runs over all gyroscope and accelerometer axes of SINS1 and SINS2 (six gyro axes and four accelerometer axes in total). This improvement ratio measures the fraction of the total bias that has been effectively compensated by the proposed method. Table 4 summarizes the bias estimation error statistics and their corresponding improvement ratios for both SINSs after filter convergence (after 2 h).

The bias estimation results show that the bias values of the SINSs used in the experiments are significantly larger than those in the simulation settings, especially the gyro biases, and they exhibit time-varying fluctuations due to both inherent sensor characteristics and environmental factors. However, the navigation error results in Figure 15 show that the positioning errors of the two SINSs remain comparable to those in the simulation, indicating that the proposed method is insensitive to bias variations and demonstrates a certain degree of robustness.

Similar to the simulation results, the experimental results demonstrate that with only relative range and angle measurement constraints, the common-mode errors ($\left|\delta v_{E_{1}}+\delta v_{E_{2}}\right|$, $\left|\delta v_{E_{1}}+\delta v_{E_{2}}\right|$, $\left|\delta L_{1}+\delta L_{2}\right|$, $\left|\delta \lambda_{1}+\delta \lambda_{2}\right|$) cannot become observable, so the long-term drift trends of position and velocity errors for the two SINSs remain consistent. However, visible high-frequency fluctuations can be observed on the velocity error curves. Unlike the ideal simulation environment with highly matched sensor parameters and controlled error conditions, the two physical inertial units in the experiment experience independent high-frequency disturbances, including inherent sensor noise, asynchronous time-varying bias fluctuations, and environmental perturbations during operation. Since velocity error directly reflects the instantaneous effect of such high-frequency difference-mode disturbances, the burr-like discrepancies between the two SINSs are clearly distinguishable.

In contrast, position error is the time integral of velocity error, which inherently attenuates the amplitude of high-frequency components. In the full-scale view dominated by hundred-meter-level long-term drift, these meter-level high-frequency burrs are visually submerged. This does not contradict the observability conclusion—the core unobservable low-frequency common-mode drift still dominates the long-term error evolution of both SINSs.

**Figure 16:**
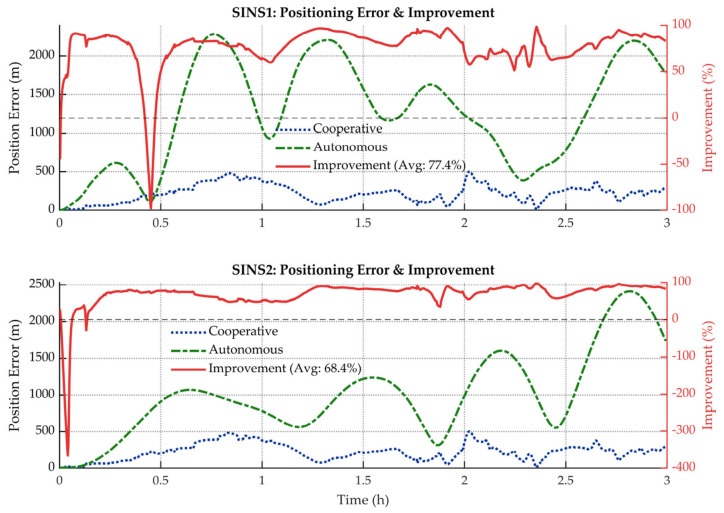
Field experiment results: positioning errors of autonomous and cooperative navigation modes.

**Figure 17:**
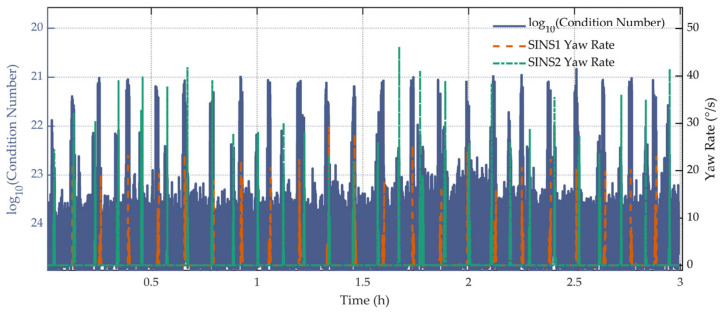
Field experiment results: effect of turning maneuvers on system observability.

Figure 16 and Figure 17 present the navigation error estimation results and the sliding-window (30 s) FIM condition numbers, respectively. During the 3 h period of cooperative navigation, the average positioning accuracy improvements are 77.4% for SINS1 and 68.4% for SINS2, corresponding to greater enhancement for the lower-precision SINS. In Figure 17, the intervals of reduced condition number align closely with the turning maneuvers, and the condition number during these segments is approximately two orders of magnitude lower than that during uniform-motion or stationary periods. This demonstrates that turning maneuvers effectively improve system observability even under realistic measurement noise and motion deviations, corroborating the observability analysis.

In summary, the experimental results demonstrate that under the experimental conditions described, turning maneuvers reduce the FIM condition number by approximately two orders of magnitude, which improves the observability of key error parameters and enables accurate estimation of all gyroscope and accelerometer biases. Consequently, the overall positioning accuracy of the two SINSs improves significantly: the RLG-50-INS improves by 77.4%, while the RLG-90-INS improves by 68.4% over the autonomous navigation mode. These results validate the feasibility of the proposed method in practical engineering applications.

## 6. Conclusions

In order to address the problem of navigation error divergence of SINSs in GNSS-denied environments, this paper proposes a cooperative navigation method aided by relative range and angle measurements to enhance the absolute positioning accuracy of dual-SINS cooperative system. By establishing a relative measurement constraint model between two SINSs, the proposed method achieves online estimation of all biases using only planar motion without relying on any external absolute reference, effectively suppressing navigation error divergence. Field experiments are conducted to verify the robustness and effectiveness of the proposed method.

The experimental results show that with relative range and angle measurement constraints, the biases of each axis can converge within 1–2 h when the two SINSs perform cooperative navigation along a figure-8 trajectory. The average positioning accuracy within 3 h is improved by more than 68% compared with autonomous navigation, with even greater improvement for medium- and low-precision SINSs. The proposed method requires only two SINSs and relatively simple planar motion, without the need for reference benchmarks, complex three-dimensional excitation trajectories, or turntable modulation. It reduces system complexity and motion requirements, providing an effective and easy-to-implement solution for cooperative navigation in GNSS-denied environments.

Future work will focus on extending the proposed method to multi-platform (N > 2) scenarios and on developing observability improvement strategies under degraded relative measurement conditions, such as range-only measurements, to accommodate more complex practical application requirements.

## Figures and Tables

**Table 1:** Coordinate frames.

Symbol	Description
i	Earth-centered inertial coordinate frame, with origin at the Earth’s center, z-axis aligned with Earth’s rotation axis and x-axis pointing to the vernal equinox.
e	Earth-centered Earth-fixed coordinate frame, with origin at the Earth’s center, rotating with the Earth and x-axis pointing to the prime meridian-equator intersection.
n	Navigation coordinate frame, with its x, y, z axes pointing to the east, north and up directions, respectively.
$n^{\prime }$	Computational navigation coordinate frame with misalignment relative to the n-frame.
b	SINS body coordinate frame, with its x, y, z axes aligned with the right, forward and upward directions of the vehicle body, respectively.
o	RAMD measurement coordinate frame, with its x, y, z axes pointing to the right, forward and upward directions of the RAMD, respectively.

**Table 2:** Error parameters of SINSs.

Error Parameters	SINS1	SINS2
Gyroscope Bias	0.005°/h	0.01°/h
Gyroscope Random Walk	$0.0005{}^{\circ}/\sqrt{h}$	$0.0005{}^{\circ}/\sqrt{h}$
Accelerometer Bias	50 mGal	80 mGal
Accelerometer Random Walk	$10\;\mathrm{mGal}/\sqrt{\mathrm{Hz}}$	$10\;\mathrm{mGal}/\sqrt{\mathrm{Hz}}$

**Table 3:** Device specifications in the field experiment.

Instruments	Parameters	Accuracy	Frequency
RLG-50	Bias stability	≤0.01°/h	100 Hz
	Random walk	$0.002{}^{\circ}/\sqrt{h}$	
RLG-90	Bias stability	≤0.003°/h	
	Random walk	$0.0005{}^{\circ}/\sqrt{h}$	
Accelerometer	Bias stability	≤20 mGal	
	Random walk	$10\;\mathrm{mGal}/\sqrt{\mathrm{Hz}}$	
GNSS receiver	Position	2 m	1 Hz
RH-6 electro-optical pod	Position	1% of relative distance	
	Angle	$1^{\prime }$	

**Table 4:** Bias estimation error statistics for cooperative navigation.

Parameters	Maximum Error	RMSE	Improvement (%)
$\varepsilon_{x}^{b_{1}}/\left({}^{\circ}/h\right)$	$6.36\times 10^{-3}$	$5.04\times 10^{-3}$	89.62
$\varepsilon_{y}^{b_{1}}/\left({}^{\circ}/h\right)$	$2.58\times 10^{-3}$	$1.72\times 10^{-3}$	95.71
$\varepsilon_{z}^{b_{1}}/\left({}^{\circ}/h\right)$	$6.80\times 10^{-3}$	$3.24\times 10^{-3}$	79.42
$\varepsilon_{x}^{b_{2}}/\left({}^{\circ}/h\right)$	$5.82\times 10^{-3}$	$3.81\times 10^{-3}$	82.16
$\varepsilon_{y}^{b_{2}}/\left({}^{\circ}/h\right)$	$9.99\times 10^{-3}$	$9.40\times 10^{-3}$	75.49
$\varepsilon_{z}^{b_{2}}/\left({}^{\circ}/h\right)$	$9.39\times 10^{-3}$	$4.28\times 10^{-3}$	59.82
$\nabla_{x}^{b_{1}}/\mathrm{mGal}$	13.64	12.35	95.90
$\nabla_{y}^{b_{1}}/\mathrm{mGal}$	34.83	33.30	81.13
$\nabla_{x}^{b_{2}}/\mathrm{mGal}$	10.30	6.64	83.45
$\nabla_{y}^{b_{2}}/\mathrm{mGal}$	5.97	3.83	31.39

## Data Availability

The raw data supporting the conclusions of this article will be made available by the authors on request.

## Appendix A

### Appendix A.1. Derivation of the Attitude Error Equation

Given ${\tilde{C}}_{b}^{n}=\left(I-\left[\phi^{n}\times \right]\right)C_{b}^{n}$, ${\tilde{\omega }}_{in}^{n}=\omega_{in}^{n}+\delta \omega_{in}^{n}$, ${\tilde{\omega }}_{ib}^{b}=\omega_{ib}^{b}+\delta \omega_{ib}^{b}$, and neglecting second-order error terms:

(A1) $$\begin{array}{cc} {\dot{\tilde{C}}}_{b}^{n} & ={\tilde{C}}_{b}^{n}\left[{\tilde{\omega }}_{ib}^{b}\times \right]-\left[{\tilde{\omega }}_{in}^{n}\times \right]{\tilde{C}}_{b}^{n}\\ \; & =\left(I-\left[\phi^{n}\times \right]\right)C_{b}^{n}\left[\left(\omega_{ib}^{b}+\delta \omega_{ib}^{b}\right)\times \right]-\left[\left(\omega_{in}^{n}+\delta \omega_{in}^{n}\right)\times \right]\left(I-\left[\phi^{n}\times \right]\right)C_{b}^{n} \end{array}$$

(A2) $$\begin{array}{cc} {\dot{\tilde{C}}}_{b}^{n} & =\frac{d}{dt}\left[\left(I-\left[\phi^{n}\times \right]\right)C_{b}^{n}\right]\\ \; & =-\left[{\dot{\phi }}^{n}\times \right]C_{b}^{n}+\left(I-\left[\phi^{n}\times \right]\right)\left(C_{b}^{n}\left[\omega_{ib}^{b}\times \right]-\left[\omega_{in}^{n}\times \right]C_{b}^{n}\right) \end{array}$$

By equating the two expressions above, right-multiplying both sides by $C_{n}^{b}$ and simplifying via skew-symmetric matrix properties, the attitude error equation is derived as:

(A3) $${\dot{\phi }}^{n}=-\omega_{in}^{n}\times \phi^{n}+\delta \omega_{in}^{n}-C_{b}^{n}\delta \omega_{ib}^{b}$$

### Appendix A.2. Derivation of the Velocity Error Equation

Given ${\tilde{f}}^{b}=f^{b}+\delta f^{b}$, ${\tilde{\omega }}_{ie}^{n}=\omega_{ie}^{n}+\delta \omega_{ie}^{n}$, ${\tilde{\omega }}_{en}^{n}=\omega_{en}^{n}+\delta \omega_{en}^{n}$, ${\tilde{v}}^{n}=v^{n}+\delta v^{n}$ and neglecting second-order error terms:

(A4) $$\begin{array}{l} \delta {\dot{v}}^{n}={\dot{\tilde{v}}}^{n}-{\dot{v}}^{n}\\ ={\tilde{C}}_{b}^{n}\left({\tilde{f}}^{b}\right)-\left(2{\tilde{\omega }}_{ie}^{n}+{\tilde{\omega }}_{en}^{n}\right)\times {\tilde{v}}^{n}+g^{n}-\left[C_{b}^{n}f^{b}-\left(2\omega_{ie}^{n}+\omega_{en}^{n}\right)\times v^{n}+g^{n}\right]\\ =\left[C_{b}^{n}f^{b}\times \right]\phi^{n}-\left(2\omega_{ie}^{n}+\omega_{en}^{n}\right)\times \delta v^{n}-\left(2\delta \omega_{ie}^{n}+\delta \omega_{\mathrm{en}}^{n}\right)\times v^{n}+C_{b}^{n}\delta f^{b} \end{array}$$

### Appendix A.3. Derivation of the Position Error Equation

Given $\tilde{L}=L+\delta L$, $\tilde{\lambda }=\lambda +\delta \lambda$ and neglecting second-order error terms:

(A5) $$\delta \dot{L}=\dot{\tilde{L}}-\dot{L}=\frac{v_{N}+\delta v_{N}}{R_{N}+h}-\frac{v_{N}}{R_{N}+h}=\frac{\delta v_{N}}{R_{N}+h}$$

(A6) $$\delta \dot{\lambda }=\dot{\tilde{\lambda }}-\dot{\lambda }=\frac{\sec\left(L+\delta L\right)\left(v_{E}+\delta v_{E}\right)}{R_{E}+h}-\frac{v_{E}\sec L}{R_{E}+h}=\frac{v_{E}\tan L\sec L}{R_{E}+h}\delta L+\frac{\sec L}{R_{E}+h}\delta v_{E}$$

## Appendix B

The Parameter Matrix M in $F_{1}\left(t\right)$ and $F_{2}\left(t\right)$

$$\begin{array}{l} M_{13}=\left[\begin{array}{ccc} 2v_{N_{1,2}}\omega_{\mathrm{ie}}\cos L_{1,2}+\frac{v_{E_{1,2}}v_{N_{1,2}}}{\left(R_{E}+h_{1,2}\right)\cos^{2}L_{1,2}} & 0 & \frac{-v_{E_{1,2}}v_{N_{1,2}}\tan L_{1,2}}{{\left(R_{E}+h_{1,2}\right)}^{2}}\\ -2v_{E_{1,2}}\omega_{\mathrm{ie}}\cos L_{1,2}-\frac{v_{E_{1,2}}^{2}}{\left(R_{E}+h_{1,2}\right)\cos^{2}L_{1,2}} & 0 & \frac{v_{E_{1,2}}^{2}\tan L_{1,2}}{{\left(R_{E}+h_{1,2}\right)}^{2}}\\ -2v_{E_{1,2}}\omega_{\mathrm{ie}}\sin L_{1,2} & 0 & -\frac{v_{N_{1,2}}^{2}}{{\left(R_{N}+h_{1,2}\right)}^{2}}-\frac{v_{E_{1,2}}^{2}}{{\left(R_{E}+h_{1,2}\right)}^{2}} \end{array}\right]\\ M_{21}=\left[\begin{array}{ccc} 0 & -\frac{1}{R_{N}+h_{1,2}} & 0\\ \frac{1}{R_{E}+h_{1,2}} & 0 & 0\\ \frac{\tan L_{1,2}}{R_{E}+h_{1,2}} & 0 & 0 \end{array}\right],\;M_{23}=\left[\begin{array}{ccc} 0 & 0 & \frac{v_{N_{1,2}}}{{\left(R_{N}+h_{1,2}\right)}^{2}}\\ -\omega_{\mathrm{ie}}\sin L_{1,2} & 0 & \frac{v_{E_{1,2}}}{{\left(R_{E}+h_{1,2}\right)}^{2}}\\ \omega_{\mathrm{ie}}\cos L_{1,2}+\frac{v_{E_{1,2}}}{\left(R_{E}+h_{1,2}\right)\cos^{2}L_{1,2}} & 0 & \frac{v_{E_{1,2}}\tan L_{1,2}}{{\left(R_{E}+h_{1,2}\right)}^{2}} \end{array}\right]\\ M_{31}=\left[\begin{array}{ccc} 0 & \frac{1}{R_{N}+h_{1,2}} & 0\\ \frac{1}{\left(R_{E}+h_{1,2}\right)\cos L_{1,2}} & 0 & 0\\ 0 & 0 & 1 \end{array}\right],\;M_{32}=\left[\begin{array}{ccc} 0 & 0 & -\frac{v_{N_{1,2}}}{{\left(R_{N}+h_{1,2}\right)}^{2}}\\ \frac{v_{E_{1,2}}\sin L_{1,2}}{\left(R_{E}+h_{1,2}\right)\cos^{2}L_{1,2}} & 0 & -\frac{v_{E_{1,2}}}{{\left(R_{E}+h_{1,2}\right)}^{2}\cos L_{1,2}}\\ 0 & 0 & 0 \end{array}\right] \end{array}$$
